# Predictive clinical model of tumor response after chemoradiation in rectal cancer

**DOI:** 10.18632/oncotarget.19651

**Published:** 2017-07-28

**Authors:** Marisa D Santos, Cristina Silva, Anabela Rocha, Carlos Nogueira, Fernando Castro-Poças, António Araujo, Eduarda Matos, Carina Pereira, Rui Medeiros, Carlos Lopes

**Affiliations:** ^1^ Department of Surgery, Digestive Surgery Service, Hospital Center of Porto, Porto, Portugal; ^2^ Abel Salazar Biomedical Science Institute, University of Porto, Porto, Portugal; ^3^ Gastroenterology Service, Hospital Center of Porto, Porto, Portugal; ^4^ Service of Medical Oncology, Hospital Center of Porto, Porto, Portugal; ^5^ Department of Health Community, Abel Salazar Biomedical Science Institute, University of Porto, Porto, Portugal; ^6^ Molecular Oncology and Viral Pathology Group, IPO Research Center, Portuguese Oncologic Institute, Porto, Portugal; ^7^ Research Department, Portuguese League Against Cancer, Porto, Portugal; ^8^ CEBIMED, Faculty of Health Sciences of Fernando Pessoa, University of Porto, Porto, Portugal; ^9^ Department of Pathology, Pathological Anatomy Service, Hospital Center of Porto, Porto, Portugal; ^10^ Department of Pathology and Molecular Immunology, Abel Salazar Biomedical Science Institute, University of Porto, Porto, Portugal

**Keywords:** neutrophil lymphocyte ratio, molecular marker, neoadjuvant chemoradiation, prediction, rectal cancer

## Abstract

Survival improvement in rectal cancer treated with neoadjuvant chemoradiotherapy (nCRT) is achieved only if pathological response occurs. Mandard tumor regression grade (TRG) proved to be a valid system to measure nCRT response. The ability to predict tumor response before treatment may significantly have impact the selection of patients for nCRT in rectal cancer. The aim is to identify potential predictive pretreatment factors for Mandard response and build a clinical predictive model design. 167 patients with locally advanced rectal cancer were treated with nCRT and curative surgery. Blood cell counts in peripheral blood were analyzed. Pretreatment biopsies expression of cyclin D1, epidermal growth factor receptor (EGFR), vascular endothelial growth factor (VEGF) and protein 21 were assessed. A total of 61 single nucleotide polymorphisms were characterized using the Sequenom platform through multiplex amplification followed by mass-spectometric product separation. Surgical specimens were classified according to Mandard TRG. The patients were divided as: “good responders” (Mandard TRG1-2) and “poor responders” (Mandard TGR3-5). We examined predictive factors for Mandard response and performed statistical analysis. In univariate analysis, distance from anal verge, neutrophil lymphocyte ratio (NLR), cyclin D1, VEGF, EGFR, protein 21 and rs1810871 interleukin 10 *(IL10)* gene polymorphism are the pretreatment variables with predictive value for Mandard response. In multivariable analysis, NLR, cyclin D1, protein 21 and rs1800871 in *IL10* gene maintain predictive value, allowing a clinical model design. Conclusion: It seems possible to use pretreatment expression of blood and tissue biomarkers, and build a model of tumor response prediction to neoadjuvant chemoradiation in rectal cancer.

## INTRODUCTION

Colorectal cancer is still one of the most common malignancy in Western Countries and the second in mortality, despite all improvements in therapeutic approach [1]. Treatment in locally advanced rectal cancer (LARC) is paradigmatic for this issue. Neoadjuvant chemoradiotherapy (nCRT) combined with surgery remains the standard treatment strategy for patients with LARC, but not all patient benefits with this kind of multimodal treatment. The responses to nCRT, range from none to complete, and only patients with complete or near complete response have improved local recurrence control and disease free survival [2–7]. In fact, the variety of tumor responses increased the need to find a useful predictive model, which may be helpful in the design of individualized treatment for rectal cancer. However, the accuracy of the current available imaging modalities, including diffusion-weighted magnetic resonance imaging (MRI) and positron emission tomography (PET) on restaging tumor after nCRT seems to be less effective than was expected to be [8–10]. For this reason, the search for biomarkers predictors of rectal cancer response to chemoradiation, retains all relevance and great interest [11, 12].

Several promising candidate markers have been reported as potential roles to predict nCRT response. In fact, host, tumor biology, and treatment-related factors may affect nCRT response and interfere with outcome results in LARC. Single nucleotide polymorphisms (SNPs), a form of study patient genetic variability, hold considerable promise to unveil the underlying complex genetics of response to CRT [13–16]. Blood cell counts in peripheral blood are considered to reflect environmental host factors in rectal tumor and may be correlated with survival and even tumor response [17–19]. CEA level, tumor size, distance from anal verge, differentiation grade, as well molecular features, are pretreatment tumor aspects that can be related with tumor response [20–27]. Between these aspects, molecular features assume particular relevance reflecting the tumor biology. In this area, multiple attempts by individual molecular markers in tissue microarray studies have emerged with the goal of identifying predictors of a response to chemoradiation in patients with rectal cancer [28, 29]. The expression of several biomarkers in pretreatment biopsies, such cyclin D1, p21, EGFR and VEGF have been associated with tumor response to nCRT. On the other hand, chemoradiotherapy scheme selection and interval of time between nCRT and surgery are treatment-related factors that can constrain nCRT response magnitude [30–33].

Our objective was to assess whether neutrophil lymphocyte ratio (NLR) in pretreatment blood sample, pretreatment biopsies expression of cyclin D1, EGFR, VEGF and p21, and 61 tagSNPs (genomic DNA was extracted from peripheral blood leukocytes or tumor tissue) could be used to predict pathologic response in the setting of rectal cancer treated in a single tertiary center with nCRT followed by surgery. Mandard TRG were measured and used to define tumor response. The patients were divided as: “good responders” (Mandard TRG1-2) and “poor responders” (Mandard TRG3- 5). According to the results from our previous data, good responders have better prognosis than poor responders [34, 35]

## RESULTS

### Description of study population and clinical parameters

This cohort study gathered 186 consecutive patients with LARC treated with nCRT follow by curative surgery with total mesorectal excision at one single University Hospital. After the exclusion of 11 patients with positive radial margin (R1 surgery), for 4 patients with yp stage IV, and four deaths within 60 postoperative days, 167 patients were included in the present analysis, with a median age of 64.6 years (range = 29-83 years). The male to female ratio was 1.69:1. The clinical parameters are summarized in Table 1. Description of Study Population

**Table 1:** Clinical parameters of patients included in this study

Variable	Value/N (%)
Age, years Mean Range	64.62 (29-83)
Gender Male Female	105 (62.9) 62 (37.1)
Tumor length < 4 cm 4-6 cm ≥ 6 cm	41 (24.6) 85 (50.8) 41 (24.6)
Tumor luminal circumference extension ≤ 1/3 > 1/3 and ≤ 1/2 > 1/2 and ≤ 2/3 > 2/3 and ≤ 3/3	27 (16.2) 59 (35.3) 39 (23.4) 42 (25.1)
Distance from anal verge > 6 cm ≤ 6 cm	81 (48.5) 86 (51.5)
Pre‐CRT CEA < 5 ng/ml ≥ 5 ng/ml Missing	116 (69.5) 44 (26.3) 7 (4.2)
NLR < 3 **≥** 3	96 (57.4) 71 (42.5)
cT stage 2-3 4	152 (91.0) 15 (9.0)
Clinical stage II III	76 (45.5) 91 (54.5)
Post‐CRT CEA < 5 ng/ml ≥ 5 ng/ml Missing data	141 (84.4) 14 (8.4) 12 (7.2)
Surgical procedure AAR/SSO APR /other	107 (64.1) 60 (35.9)
Surgery Open Laparoscopic	129 (77.2) 38 (22.8)
Perioperative complications Morbidity Abdominal or pelvic abscess Anastomose leak Reoperation Re-admission	42 (25.1) 16 (9.5) 3 (1.7) 6 (3.5) 3 (1.7)

Pre-CRT CEA: pre-chemoradiotherapy carcinoembryonic antigen; NLR: neutrophil lymphocyte ratio. cT stage: clinical T stage of TNM staging system; AAR: anterior abdominal resection; SSO: sphincter-saving operation; APR: abdominoperineal resection.

### Polymorphism study

Three SNPs were excluded from the analysis due to genotyping failure, leaving a total of 58 to be included in the survival analysis: 29 in *ABCC4*, 15 in *SLCO2A1*, seven in *HPGD*, and one each in *COX2, EGFR, CCND1, IL10, TNFA, IGF1* and *NAT2* genes. Description, frequencies and predictive response value of selected SNPs in the 167 patients are given in Supplementary Table S1b.

### Biopsy characteristics

The biopsy characteristics including immunohistochemical expression of cyclin D1, p21, EGFR and VEGF are shown in Table 2.

**Table 2:** Biopsy characteristics

Variable	N (%)
Grade 1 2 3 Missing data	46 (27.5) 98 (58.7) 8 (4.8) 15 (9.0)
Mucinous presence No Yes Missing data/not applied	145 (86.8) 7 (4.2) 15 (9.0)
Mitosis number ≤ 9,5 ≥ 9,6 Missing data/not applied	34 (20.4) 117 (70.0) 16 (9.6)
Inflammatory infiltrate Scarce Moderate Marked Missing data/not applied	33 (19.8) 66 (39.5) 52 (31.1) 16 (9.6)
Desmoplastic reaction Scarce Moderate Marked Missing data/not applied	44 (26.3) 80 (47.9) 27 (16.2) 16 (9.6)
Degree of necrosis Scarce Moderate Marked Missing data/not applied	79 (47.3) 40 (23.9) 32 (19.2) 16 (9.6)
IHC study of biopsy Cyclin D1 Weak ( ≤ 3) Strong (>3) Missing EGFR Weak (≤<3) Strong (>3) VEGF Weak (≤3) Strong (>3) p21 Weak (≤3) Strong (>3)	126 (75.4) 38 (22.7) 3 (1.7) 89 (53.2) 78 (46.7) 58 (34.7) 109 (65.2) 100 (59.8) 67 (40.1)

EGFR: epidermal grow factor receptor; VEGF: vascular endothelial growth factor; p21: p21 protein or cyclin-dependent kinase inhibitor or CDK-interacting protein 1

### Surgery

Sphincter-saving rectal resection with anastomosis (with or without protective ileostomy) was performed on 107 patients (64.1%). Abdominoperineal resection was performed on 53 patients, and seven patients were subjected to proctectomy with definitive stoma. The perioperative morbidity of the series was 25.1%, with 16 abdominal or pelvic abscesses, three anastomostic leaks, that included six reoperations (due to three leakages and three abdominal abscesses), and three re-admissions (due to pelvic abscess).

### Pathology of resected specimens

Stage distribution is shown in Table 3.

**Table 3:** Characteristics of resected specimens

Variable	N (%)
ypT stage pT0-1 pT2-4	38 (22,8) 129 (77.2)
ypN stage pN0 pN1-2	110 (65.9) 57 (34.1)
Pathological stage 0 II III	58 (34.7) 58 (34.7) 51 (30.5)
T downstaging Yes No	67 (40.1) 100 (59.9)
Pathological TNM downstaging Yes No	95 (56.9) 72 (43.1)
CRM distance > 2mm ≤ 2 and >1mm	159 (95.2) 8 (4.8)
Distal margin ≥ 2cm < 2 cm and ≥1 cm	108 (65.7) 59 (35.3)
Mandard TRG TRG 1-2 (Good responders) TRG 3-5 (Poor responders)	86 (51.4) 81 (48.5)
Linfatic permeation No Yes	100 (59.9) 67 (40.1)
Vascular permeation No Yes	142 (85) 25 (15)
Perineural permeation No Yes	96 (57.5) 71 (42.5)
Grade 0 1 2 3	32 (19.2) 31 (18.6) 98 (58.7) 6 (3.6)
Mucinous presence Yes No	41 (24.5) 126 (75.4)
Inflammatory infiltrate Scarce Moderate Marked Missing data	60 (35.9) 75 (44.9) 28 (16.8) 4 (2.4)
Desmoplastic reaction Scarce Moderate Marked Missing data	45 (26.9) 64 (38.3) 51 (30.5) 7 (4.2)
Necrosis grade Scarce Moderate Marked Missing	27 (16.2) 38 (22.8) 99 (59.3) 3 (1.8)
Mitotic number ≤ 9.5 > 9.5 Missing data	73 (43.7) 68 (40.7) 26(15.6)
IHC study Cyclin D1 Weak ( ≤ 3) Strong (>3) Missing data EGFR Weak (≤<3) Strong (>3) VEGF Weak (≤3) Strong (>3) p21 Weak (≤3) Strong (>3)	143 (85.6) 32 (13.8) 1 (0.6) 97 (58.1) 70 (41.9) 46 (27.5) 121 (72.5) 110 (65.9) 57 (43.1)

ypT Stage: Pathological postneoadjuvant therapy T stage of TMN staging system; ypN stage: pathological postneoadjuvant therapy N stage of of TMN staging system; CRM: circumferential radial margin; Mandard TRG: Mandard tumor regression grades; IHC study: immunohistochemistry; EGFR: epidermal growth factor receptor; VEGF: vascular endothelial growth factor; p21: p21 protein.

The average number of dissected lymph nodes in the surgical specimens was 8 (range = 0-22). Circumferential resection margin > 1 mm was confirmed in all 167 patients.

Response to neoadjuvant therapy is characterized in Table 3. Tumor downstaging was observed in 67 patients (40.1%).

Reduction in T-stage by one level was observed in 29 patients (17.4%) and by two or more levels in 38 patients (22.8%). Observations indicating pathological downstaging are given in Table 3. Ninety five (56.8%) patients presented one or lower pathological stage than their initial clinical tumor stage. ypCR or Mandard TRG1 was confirmed in 31 patients (18.5%). The use of Mandard system allowed us to define two groups as previously mentioned: good responders (Mandard TRG1-2) and poor responders (Mandard TRG3- 5). Using the Mandard system, a good response to nCRT was found in 86 patients (51.4%) and a poor response in 81 (48.5%).

### Clinical outcome

Table 4 shows long-term clinical outcome, relapse of disease and survival. With a median follow-up of 64 months (range = 6-148 months), 5-year DFS was 73.3% and pelvic control was 95.8%. Seven patients (4.2%) developed pelvic recurrence (five isolated and two with synchronous metastatic disease) and 22 (13.2%) distant metastases alone. Two of the seven pelvic recurrences occurred later in the outcome - after 18 months of follow-up (at 28 and 45 months, respectively). Twelve out of 167 patients had more than 24 but less than 42 months of follow-up. Local recurrence increased slightly to 4.5% (7/155) when considering only patients with more than 42 months of follow-up.

**Table 4:** Long-term clinical outcome, relapse of disease and overall survival (DFS)

Variable	
** Overall disease recurrence** Local Distant Local and distant	29 (17.3%) 5 (3%) 22(13.2%) 2 (1.1%)
**Five year overall survival**	74,6% (se=3.8%)
**Five year overall survival for “good responders” Five year overall survival for “poor responders”**	88,3% (se=3.9%) p<0.001 (log Rank test) * 58,3% (se=6.5%) Cox model survival HR= 5,16 C.I. 95% (2,638-10,114)

* Significantly different at *p* < 0.001 (log-rank test); se: standard error.

Using results from a fitted Cox model, the estimated hazard ratio is 5.16: this means that the poor responders have a probability of death five times higher than good responders ( Cox model survival HR = 5,16 C.I. 95% 2,638-10,114).

### Predictive factors of Mandard response in pretreatment variables

A logistic regression analysis was used to assess the independent significance of pretreatment variables as predictive factors of Mandard poor response (TRG3-5).

In this study, all variables were analyzed before neoadjuvant treatment, once our aim is to predict tumor response before beginning CRT.

In polymorphisms study, only one genetic polymorphism (rs1800781) in *IL10* gene has a predictive response value. The others pretreatment variables with significant predictive value are anal tumor distance, cyclin D1, p21, EGFR and VEGF in biopsies and neutrophil lymphocyte ratio in blood samples. The remaining variables of Tables 1, 2 have no significant predictive value.

Table 5 shows these seven variables with predictive value for Mandard response in univariate analysis.

**Table 5:** Predictive value of pretreatment variables to Mandard response (Univariate analysis of logistic regression: dependent variable Mandard good response – 0; Mandard poor response – 1) C.I. 95%

Variable	N	Poor response %	p	OR	C.I. 95%
Anal tumor distance ≤6 cm >6 cm	86 81	40.7 58.0	0.025	1 2.014	1.00 ‐3.730
NL Ratio <3 ≥3	96 71	39.6 62.0	0.004	1 2.487	1.325 – 4.670
Cyclin D1 Weak (≤3) Strong (>3)	126 38	42.1 71.1	0.002	1 3.381	1.542 – 7.141
p21 Weak (≤3) Strong (>3)	100 67	34.0 71.6	<0.001	1 4.904	2.501 9.616
EGFR Weak (≤3) Strong (>3)	89 78	39.33 60.3	0.007	1 2.339	1.256 – 4.356
VEGF Weak (≤3) Strong (>3)	58 109	43.1 52.3	0.26 ns	1 1.447	0.762 – 2.748
rs1800871 AG GG AA	67 80 15	55.2 38.8 66.7	0.048 0.047 0.421 ns	1 0.513 1.622	0.265 ‐ 0.991 0.500 – 5.259

NL ratio: neutrophil lymphocyte ratio; p21: p21 protein; EGFR: epidermal growth factor receptor; VEGF: vascular endothelial growth factor; rs 1800871 single nucleotide polymorphism of interleukin 10 (*IL 10)* gene

The seven variables were entered as covariates into the logistic regression ( method: forward likelihood ratio). This selection was based upon their statistical significance in univariate analysis and their potential clinical interaction. In the multivariate analysis, after adjustment for the effects of the other variables, NLR, Cyclin D1, p21 and rs1800871 in *IL10* gene were more likely to present a poor response (Table 6).

**Table 6:** Multivariate stepwise forward analysis LR; dependent variable – Mandard response (good response = 0; poor response = 1)

	B	S.E.	Sig	Exp(B)	C.I. 95%EXP(B)	
					Lower	Upper
NLR ≥ 3	1,106	0,395	0,005	3,022	1,394	6,554
Cyclin D1 (>3)	1,436	0,469	0,002	4,206	2,761	11,103
p 21 (>3)	1,755	0,393	0,0001	5,784	2,676	12,503
rs1800871 AG			0,024			
rs1800871 GG	- 0,441	0,394	0,264	0,644	0,297	1,395
rs1800871 AA	1,425	0,704	0,043	4,160	1,046	16,545
Constant	‐1,487	0,414	0,0001	0,226		

NL ratio: neutrophil lymphocyte ratio; p21: p21 protein; rs 1800871: single nucleotide polymorphism of interleukin 10 (*IL 10)* gene

Estimated logistic probability of Mandard response = e^g(ˣ)^ / 1 + e^g(ˣ)^

Considering the equation of the logistic probability model obtained from the Table 6: e = e^g^(ˣ) / 1 + e^g^(ˣ), we can calculate the probability of a patient with a poor Mandard response.

For example, if the patient has the best scores:

g(x) = -1.487- 0.441 rs1800871 GG = -1.928 ;

Mandard poor response = 0.145/1+0.145 = 0.126

The probability of the patient with a Mandard poor response will be 0.126

If the patient has the worst scores :

g(x) = -1.487+1.106 NLR ≥3 + 1.436 Cyclin D1 + 1.755 p21 + 1.425 rs1800871 AA = 4.235 ;

Probability of Mandard poor response = 69.06/ 70.06 = 0.985

The probability of Mandard poor response will be 0.985

If we consider the first patient in our database:

g(x) = -1.487+ 1.106 (NLR≥3) + 1.755 (p21 > 3) - 0.441 (rs1800871GG) = 0.933 ;

Probability of poor response = 2.542 / 3.542 = 0.717

So, applying for the first patient that has a NLR > 3 and a p21 > 3 and a RS1800871GG, we would predict that it will likely have a poor Mandard response as the estimate probability of 0.72 is greater than 0.5.

## DISCUSSION

The true benefit of neoadjuvant chemoradioteraphy in LARC is achieved only when tumor response is complete or near complete [4, 5]. In those cases, tumor downsizing and downstaging, usually allows sphincter saving procedure and better local control disease and survival [36–38]. In selective cases, with complete response, surgery may be avoided and “watch and wait” policy is defensible [39, 40]. However, when tumor response is poor, during the time between the completion of CRT and surgery, we may have tumor progression. So, the prediction of tumor response before beginning CRT is a relevant issue that can affect the therapeutic strategy.

In this regard, the first difficulty is the definition and quantification of tumor response. In our previous research, Mandard tumor regression grade showed good accuracy when patients were divided as: “good responders” (Mandard TRG1-2) and “poor responders” (Mandard TRG3- 5). According to our results, good responders have better prognosis than poor responders. [34, 35, 41] This way to assess tumor response, proved to be easy, reproducible and related with survival. Others studies confirmed our findings [2–4, 42, 43] and for this reason we apply this concept to define tumor response.

The second hindrance was to find pretreatment variables with predictive value to Mandard response. Like others series, most of current pretreatment CRT clinical parameters showed no statistical significance to response prediction [3, 22, 35, 44]. In our study, from all, only tumor distance from anal verge has predictive value of Mandard response in univariate analysis who disappeared in multivariate analysis. This aspect led us to investigate host and tumor and related factors that may affect nCRT response. Three different areas were selected: inflammatory systemic markers, biomarkers IHC expression in pretreatment biopsies and polymorphisms.

Systemic inflammatory response interfere with survival in patients operated with several types of cancer, including colorectal cancer and can also affect tumor chemoradiotherapy response [18, 45]. An elevated lymphocyte count is associated with enhanced downstaging following neoadjuvant CRT for locally advanced rectal cancer [23]. However, the value of this ratio is variable in different cancers. Studies of the relationship between NLR and both survival and response to chemoradiation have been limited with respect to locally advanced rectal cancer [17, 19]. In our study, pretreatment NLR value with the cutoff of 3, based in ROC analysis, has a predictive value to Mandard response. The main advantage is the ease to measure routinely because of its low cost.

The broad and unpredictable response to tumor of patients with rectal cancer treated with neoadjuvant chemoradiation shows that our understanding of the molecular events leading to radioresistance in patients affected with this malignancy remains sparse. The expression of several biomarkers in pretreatment biopsies has been associated with tumor response to nCRT. Immunohistochemistry was the most commonly used method for detecting protein markers in tumor tissue. Epidermal growth factor receptor (EGFR), vascular endothelial growth factor (VEGF), COX-2, thymidylate synthase, the p53 tumor suppressor and key mediators of cell-cycle arrest (p21, p27) and apoptosis (Bcl-2) are among the immunohistochemical protein markers currently of interest as potential predictors of pathologic response, prognosis and recurrence-free survival in rectal cancer following neoadjuvant therapy [46–53]. We selected four - cyclin D1 and p 21 that interfere in cell cycle and are related with cell proliferation, EGFR and VEGF related with angiogenesis and tumor progression.

Cyclin D1 regulate progression from the G1 phase of the cell cycle to the S phase. As key regulators of the G1 progression step within the cell cycle, cyclin D1 may play a pivotal role in the process of carcinogenesis and cancer progression [6]. The ability of cyclin D1 to drive the cell cycle forward can be blocked by cyclin D1-dependent kinase (CDK) inhibitors, such as p27 and p21, and can explain why co-expression of cyclin D1 and p21 contributed to the role of cyclin D1 for tumor proliferation. Immunostaining for cyclin D1 was predominantly nuclear but cytoplasmic staining was detected in some cases. However, unless a nuclear staining was also detected, cases with cytoplasmic staining were considered negative. Cyclin D1 overexpressed has been reported to occur in 40-70% of colorectal tumors [54, 55]. Recently, Li et al. published a meta-analysis of observational studies that indicate Cyclin D1 overexpression, based on the nuclear staining is related with poor clinical outcome in colorectal cancer patients [56]. The cyclin D1 predictive value of tumor response value in pretreatment tumor rectal biopsies is little studied. Moral et al, between immunohistochemistry markers studies (p53, cyclin D1, Ki67, and bcl-2 protein), only found a trend towards significance for cyclin D1 [57].

p21 (Waf1/Cip1) is known as one of the cell cycle inhibitors which plays a role through the p53 dependent or independent pathway. p21 expression in tumor cells serves the cell proliferation ability and protects against DNA damage by cell cycle inhibition. p21 is a tumor suppressor gene that, once activated in response to DNA damage, causes the cell to arrest in G1 through the interruption of cyclin-dependent kinases [58]. In malignant cells, wild-type p21 suppresses apoptosis in the presence of DNA damage caused by chemotherapeutic agents or radiation [59, 60]. This may explain why high p21 expression at the pretreatment biopsy may be associated with tumor regression and poor prognosis in patients treated with 5-FU based CRT [51], but few publications have studied this aspect.

The present study showed that high cyclin D1 and high p21 expression level in the pre-CRT tumor simple were associated with poor pathologic regression (Mandard poor response). This is a concordant result considering the mechanism of action of these biomarkers.

Nevertheless, some studies show agreeing results with ours [51, 61, 62] and others don’t, probably due to the selected technique. Immunohistochemistry is highly dependent on the antibody clone that is used, staining protocols, selection of scoring methods and nuclear/cytoplasmic staining account .

EGFR is a 170-kDa transmembrane tyrosine kinase receptor that belongs to a family of receptors known as the ErbB family. EGFR is known to activate a cascade of multiple signaling pathways that facilitate tumor growth process. EGFR expression was reported to be correlated with more aggressive disease, increased risk of metastases, advanced tumor stage. Usually, IHC EGFR overexpression in tumor tissue implies worst outcome in colorectal cancer [63]. Also, Azria et al. found worst prognosis in patients with higher EGFR expression in pretreatment tumor rectal biopsies tissue [64] and Kim et al. found a predictive value of tumor downstaging absence when a high level of EGFR expression is present [46].

VEGF high expression is associated to tumor aggressiveness, poor survival, local failure and the presence of metastatic disease [65]. VEGF, is a mediator of tumor angiogenesis. New blood vessels induces increased permeability, causing less efficient delivery of chemotherapeutic agents and a decreased response to radiotherapy [66]. For this reason, VEGF assessed immunohistochemically from pretreatment tumor biopsies, may be a useful marker in the prediction of tumor response to preoperative CRT. High VEGF expression in pretreatment biopsies are correlated with poor tumor response in locally advanced rectal cancer treated with neoadjuvant chemoradiation [67]

In our study we found high expression on pretreatment biopsies of EGRF and of VEGF related with worse tumor response, but without predictive statistic value of tumor response (in univariate and multivariate analysis for VEGF and in multivariate analysis for EGRF). This result, may be related with intense staining for VEGF and EGFR pretreatment biopsies for the most of the slides.

Once again, published conflicting results can be found [68, 69]. Some of them are retrospective in nature, with a relatively small patient number and different methodologies, making it difficult to draw firm conclusions or carry out cross-study comparisons.

Despite this, in our study, cyclin D1 and p21 immunohistochemical expression in pretreatment biopsies have prediction value and are two of the four variables that contribute for the statistical model capable to predict Mandard tumor response.

The molecular markers in tissue microarray studies remains attractive: can be performed in small samples such as biopsies, the amount of commercially antibodies spent are small, making the procedure feasible and inexpensive. However, immunohistochemistry has limitations: related to reproducibility and the ability to provide quantitative information and depending on the sample representativeness, immunotyping selected and scale used. We believe that difficulties are diminished with the procedure standardization and systematic application on a large scale.

Polymorphisms were another field of interest that can help to identify Mandard good and poor responders. Polymorphisms of genes related mainly with inflammation were selected, since chronic intestinal inflammation is a well-known risk factor for colorectal cancer [70]. Cancer arises in chronically inflamed tissue. IL-10 and COX-2 are important mediators of intestinal inflammation. IL-10 is a key anti-inflammatory cytokine orchestrating the innate and adaptive immune response. Elevated cyclooxygenase-2 (COX-2) expression was found in most colorectal cancer tissue and is associated with worse survival. COX-2 plays a role in tumor progression, stimulation of angiogenesis, promotion of metastasis, and decrease of the antitumor immune response. For this reason we selected SNPs of *COX-2/HPGD/SLCO2A1/ABCC4* Prostaglandin E_2_ pathway genes and *IL10* gene among others. In our data, out of the 61 SNPs assessed, only the rs1800871 (C-592A) have a predictive value of Mandard response. The rs1800871 is a polymorphism of *IL10* gene and has been associated with colorectal cancer risk [71–73]. No previous study described a relationship between this polymorphism and tumor response to chemoradiotherapy. The main handicap of this technique is the no availability in most hospitals, being an obstacle to its application in clinical practice.

Nevertheless, with these four pretreatment variables (NLR, Cyclin D1, p-21 and rs1800871) it is possible to build a predictive clinical model of tumor response. It allows subset the patients according to the probability of response and make possible to modify the therapeutic plan for the putative “poor responders”. For example, if the patients have the best score on four variables, the probability in the model to have a “poor response” (Mandard TRG3-5) will be 12.6% (Table 6). In this group the patients nCRT is a good choice with the possibility of a good survival. On the other hand, in patients with the worst score the probability to have a “poor response” (Mandard TRG 3-5) will be 98.5% (Table 6). In this case, an alternative therapeutic may be preferable. For instance, a short course of neoadjuvant radiation instead nCRT may be a better option, with less side effects, less costs and the possibility to begin adjuvant chemotherapy sooner.

So, this predictive clinical model, in conceptual terms, is an interesting tool and can be used to decide between nCRT *versus* short course radiation, since both options do not differ in terms of survival. The advantage of our study is to had been performed in a single institution under the same criteria by a main skilled pathologist. The limitations which may have, are related to small number of the patients recruited, and the variability inter-observer that can exist in assessing of pretreatment biopsies IHC stains. Thus, the results of our study must be confirmed in regard of accuracy and reproducibility by other large and prospective studies with standard method of staining and reporting is adopted.

## CONCLUSION

It seems possible to use pretreatment expression of blood and tissue biomarkers and build a clinical model of tumor response prediction to neoadjuvant chemoradiation in rectal cancer. This clinical model may help in the therapeutic choice for locally advanced rectal cancer but, first it needs to be validated, and larger prospective clinical trials must be done, before considerer able to guide preoperative therapy choices in LARC.

## MATERIALS AND METHODS

### Ethics approval

This project was approved by the Research Ethics Health Committee (references188-CES) and Department of Education, Development and Research (reference 133-DEFI) of the Hospital Center of Porto. Informed consent from patients whose tissue was archived was not required as per Ethics Board guidelines. Patient informed consent for the use of blood and tissue for genetic polymorphism was obtained.

### Patient population

A consecutive series of patients with, biopsy-proven LARC, who underwent nCRT followed by elective radical surgery with total mesorectal excision (TME) with curative intent between January 2003 and December 2013 were recruited after reviewing the clinical database at the Digestive Surgery Service, University Hospital Center of Porto, which is a tertiary referral center for a population of 728,663 inhabitants. All patients with rectal cancer (T2N+M0 or cT3/4 N0/+M0) located at less than 12 cm of distance from the anal verge who received nCRT and were operated within 8 weeks after radiotherapy ended were included, if none of the following exclusion criteria were found: other diagnosed neoplasia, short course radiotherapy, post neoadjuvant stage IV, R1/R2 surgery, and postoperative death within 60 days.

### Diagnosis and staging criteria

Staging included rigid proctoscopy, total colonoscopy, chest, abdominal and pelvic computed tomographic scan, endorectal ultrasound, pelvic magnetic resonance image (since 2008), and serum carcinoembryonic antigen (CEA) level.

### nCRT protocol

This protocol included total irradiation of 50.4 Gy in 28 fractions and 5-fluorouracil (5-FU) by infusion pump (225 mg/m2/day, 7 days per week, from the first until the last day of radiotherapy) or capecitabine (2500 mg/m2/day, divided in two doses, from de first until the last day of radiotherapy). All patients receiving nCRT were operated on within 8 weeks after radiotherapy ended. All patients received pelvic radiation therapy with concurrent chemotherapy. The option between 5-fluorouacil and capecitabin was made based on patient comorbilities and drug tolerance.

Treatments were given with a linear accelerator with a minimum energy of 10MeV. The total dose of radiation therapy was 50.4 Gy. Patients underwent CT simulation with oral contrast to visualize the small bowel. All patients underwent three-dimensional conformal radiation treatment planning. The intent of treatment was to include the tumor bed (gross tumor volume) with a margin, the internal iliac nodes, and the presacral nodes (the external iliac nodes were also included if a structure was invaded that drained to the external iliacs) to a total dose of 45 Gy. This was delivered at 1.8 Gy per day, 5 days per week, or 25 fractions over 5 weeks. A minimum tumor boost of 540 cGy, given at 1.8 cGy per fraction, was required for all patients and was given to the gross tumor volume with a 1-cm margin. Normal tissue sparing techniques were employed so that no portion of the small bowel received more than 4500 cGy and less than 10% of the bladder receive greater than 5000 cGy.

### Surgical procedures

Radical surgery consisted mainly of sphincter-saving rectal resection or abdominoperineal resection both with total mesorectal excision. Regarding the selection of the operative procedure, we considered the distance of the lesion from the anus, the comorbidities of the patient, and the condition of the anal sphincter.

### Adjuvant chemotherapy protocol

Post surgery, patients were administered adjuvant chemotherapy protocol for 6 months performed preferably with 5-FU or a combination of 5-FU and oxaliplatin (one of the followed regimens: mFolFOX6 - 200 mg/m2 folinic acid (FA) day 1, 400 mg/m2 5-FU bolus day 1, continued infusion for 46 hours of 2400 mg/m2 5-FU and 85 mg/m2 oxaliplatin, 14/14 day cycle; CapeOx: 1000 mg/m2 capecitabine twice a day, days 1-14, 130 mg/m2 oxaliplatin day 1, 21/21 day cycle; 5-FU/FA: 200 mg/m2 FA day 1, 400 mg/m2 5-FU bolus day 1, continued infusion for 46 hours of 2400 mg/m2 5-FU, 14/14 day cycle).

mFolFOX6 or capeOX were the preferred regimens. When the administration of oxaliplatin is not possible due to side effects of the drug or the comorbidities of the patient, one of followed regimens was used: 5-fluorouracil/folinic acid (5-FU/FA) 200mg/m2 folinic acid (1-hour infusion prior to 5-FU) and 400mg/m2 5-FU per day intravenously once daily x 5 every 5 weeks or 1000 mg/m2 capecitabine twice a day x 5 every 5 weeks.

### Systemic inflammatory markers

Blood samples from all patients were collected within 7 days before starting nCRT protocol. White blood cell, neutrophil, lymphocyte, and platelet counts were recorded. The neutrophil to lymphocyte ratio (NLR) was calculated as the neutrophil count divided by the lymphocyte count using preoperative blood test results. An NLR ≥3 was considered elevated (based on receiver operating characteristic curve analysis).

### Pretreatment biopsy samples - hematoxylin and eosin (H&E) staining and immunohistochemistry (IHC)

Diagnostic pretreatment paraffin-embedded biopsies were available and reviewed by a pathologist blinded to clinical data. Tumor biopsy sample classification, including grade, were obtained for the worst tumor areas whenever available material contained several areas with neoplasia. The mitotic index was the number of mitoses for 10 high-powered fields and the cut-off was chosen taking into account the best ratio of sensitivity to specificity. In the absence of neoplasia in 10 high-power fields, the number of mitoses was counted in the number of observed fields, estimating the value for 10 fields.

IHC for cyclin D1, p21, epidermal growth factor receptor (EGFR) and vascular endothelial growth factor (VEGF) were performed in tissue arrays constructed from core tissue specimens (preoperative endoscopic biopsy) taken before treatment. Representative core tissue specimens (2 mm in diameter) were taken from individual paraffin blocks and rearranged in new tissue array blocks using a trephine apparatus (Superbiochips Laboratories, Seoul, Korea). Array slides were labeled by IHC with four commercially available antibodies to: cyclin D1 (1:1000; BD Biosciences, MA, USA), p21 (1:300; Spring Bioscience, CA, USA), EGFR (1:200; Abcam, MA, USA), and VEGF (1:30 dilution; BD Biosciences). Antigen retrieval was performed by immersing the slides in citrate buffer (pH 6.0) and microwaving them for 10 minutes. Nonreactive sites were blocked using 1% horse serum in Tris-buffered saline (pH 6.0) for 3 minutes. Primary antibodies were applied, and antibody binding was detected using the avidin-biotin peroxidase complex (Universal Elite ABC kit PK-6200; Vectastain, Burlingame, CA, USA) and diaminobenzidine tetrahydrochloride solution (Kit HK 153-5 K; Biogenex, San Ramon, CA, USA). We performed the IHC procedure without primary antibody for negative controls. Normal colorectal epithelial cells were used as internal negative controls.

**Figure 1:**
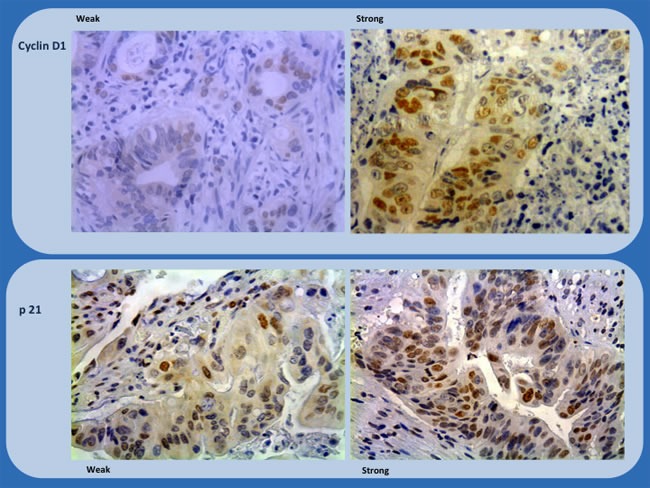
Immunohistochemistry study of biopsies (Cyclin D1 and p21) showing representative examples of weak and strong staining (x200 and x100)

**Figure 2:**
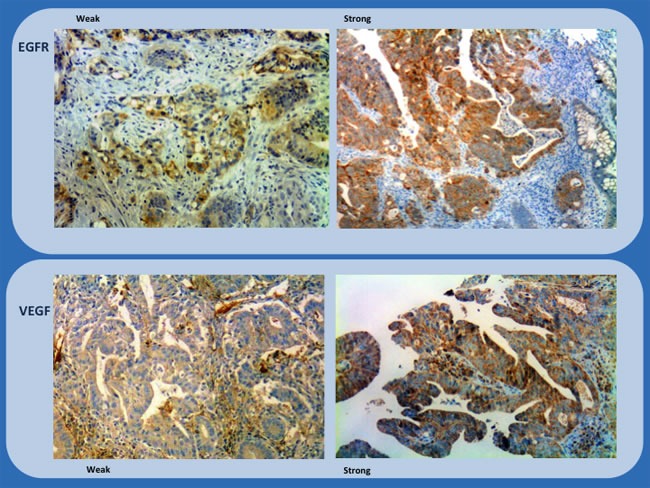
Immunohistochemistry study of biopsies (EGFR and VEGF) showing representative examples of weak and strong staining (x200 and x100)

The location of staining, nuclear, membranous, or cytoplasmic, was recorded. The percentage of positively stained cells were assigned to one of four categories for protein expression: 0%, 0; 1-25%, 1; 26-50%, 2; > 50%, 3. The staining intensity was scored as follows: none or weak, 0; moderate, 1; intense, 2. If the staining intensity was heterogeneous in a section, it was scored based on that which was predominantly observed. The two scores (if different from zero) were then multiplied to produce a weighted score for each tumor specimen (Table 7). The final score was grouped as follow 0-3, weak; ≥4, strong (Figures 1 and 2).

**Table 7:** IHC score calculation

A – Area	0	≤25		26 - 50		>50
Partial score of A	0	1		2		3
B – Intensity	absent		moderate		intense	
Partial score of B	0		1		2	
Area x Intensity (A x B)	≤ 3 (0 – 3)			≥ 4 (4 – 6)		
Final score	Weak			strong		

The samples were scored by 2 independent analysts blinded to the patients’ clinical information. Expression of each protein was quantified using a visual grading system, based on the extent of the tumor staining. The mean value for the expression of each biomarker was then calculated. Tumor staining at a level higher than the mean was considered to indicate high expression, while staining at a level lower than the mean was considered to indicate low expression. The consistency of the expression scores between the 2 independent observers was greater than 90%. In cases of disagreement, the expression level was determined by consensus.

### Sample collection and biological processing for DNA analysis

DNA was extracted preferably from blood or alternatively from tumor tissue. Blood samples were collected with a standard venipuncture technique using EDTA-containing tubes before the beginning of neoadjuvant therapeutic. Genomic DNA was extracted from peripheral blood leukocytes, using the QIAamp DNA Blood Mini Kit (Qiagen, Madrid, Spain) following the manufacturer's instructions.

For patients unable to provide a blood sample, the DNA was extracted from formalin-fixed paraffin-embedded blocks from the Pathology Department at our Hospital. Two to four 10-mm thick section were used in each extraction depending on the size of tissue area (1.5-3 cm2). Briefly, the tumor tissue specimens from each glass slide were scraped, using a clean razor blade, into a 1.5-ml microcentrifuge tube followed by centrifugation at 14000-16000 × g for 3 minutes. The tissue pellets were then rehydrated with 1 ml of absolute ethanol, followed by centrifugation at 14000-16000 × g for 3 minutes and the supernatant was discarded. This step was repeated twice. The tube was left open for 15 minutes to allow any remaining ethanol to evaporate. Further steps of DNA isolation were performed using the GRS Genomic DNA Kit. Tissue, in accordance with the manufacturer's protocol (GRiSP, Porto, Portugal).

DNA was quantified using a NanoDrop 1000 Spectrophotometer (Thermo Fisher Scientific, Wilmington, DE, USA) and stored at -20˚C until genotype examination. The DNA quality was determined by measuring the optical density (OD) 260/280 ratio.

### Polymorphism selection

The strategy for polymorphism selection has been described elsewhere [74]. Briefly, using a tagSNP approach, the genetic variants were retrieved from a set of common SNPs in the Caucasian population of HapMap project (CEU) (https://ncbi.nlm.nih.gov). The Genome Variation Server (version 7.00) was used to recover tagSNPs capturing variations (i) with a minor allelic frequency of 15% or more; (ii) within the coding region of the genes plus 2 kb upstream and downstream, (iii) with a r2 greater than 0.8, and (iv) which successfully converted to the Sequenom platform (Sequenom, San Diego, CA, USA). A total of 55 tagSNPs were analyzed in *COX2, HPGD, SLCO2A1* and *ABCC4 PGE2* pathway-related genes.

Furthermore, rs2946834 insulin-like growth factor 1 (*IGF1*), rs1801280 N-acetyltransferase 2 (*NAT2*), rs1800629 tumor necrosis factor-alpha (*TNFA*), rs9344 cyclin D1 (*CCND1*), rs2227983 *EGFR*, and rs1800871 interleukin 10 (*IL10*) polymorphisms, previously found to be associated with colorectal tumor development, were also included.

### Genotype characterization

TagSNP genotyping was performed using MassARRAY iPLEX Gold technology (Sequenom) based on multiplexed amplification followed by mass-spectrometric product separation. This technique was carried-out by the Genomic Unit, Genotyping Service, Gulbenkien Science Institute.

### Resected specimen samples - H&E staining and IHC

Immunohistochemical staining for cyclin D1, p21, EGFR and VEGF was performed in tissue arrays constructed from core tissue specimens taken from the resected specimen using the methodology applied in pretreatment tumor biopsies.

Standard pathological tumor staging of the resected specimen was performed in accordance with the guidelines of the American Joint Committee on Cancer (http://www.cancerstaging.net). The circumferential resection margin was scored as positive when cancer cells were within 1 mm of the margin. Evidence of pathologic complete response after neoadjuvant treatment (ypCR) was defined as an absence of viable adenocarcinoma in the surgical specimen or the presence of lakes of mucus without tumor cells. The histology of all surgical specimens was reviewed and confirmed by a pathologist blinded to clinical data and they were classified based on the Mandard tumor regression grading system [75].

The number of tumor samples taken from the resected specimens was variable, with a mean of six paraffin blocks per case. The methodology used was the following: five samples were taken from the area with macroscopic lesion (assuming it existed), i.e. in the same manner as dealing with a specimen from a patient who had not received neoadjuvant therapy. These included the closest macroscopic approach of the macroscopic lesion to the peritoneal surface or the mesorectal excision plane, as appropriate. If no viable tumor was identified within the initial five blocks, the whole of the remainder of any macroscopic lesion in additional blocks was included. If no viable tumor was identified within these extra blocks, another three further levels from all of these blocks were taken. If no viable tumor was identified in these sections, then complete histological tumor regression was assumed.

All obtained slides were seen and reviewed by the same experienced pathologist blinded to clinicopathological data. Items observed and registered in the biopsies were subsequently analyzed in the resected specimen and the same criteria adopted.

**Figure 3:**
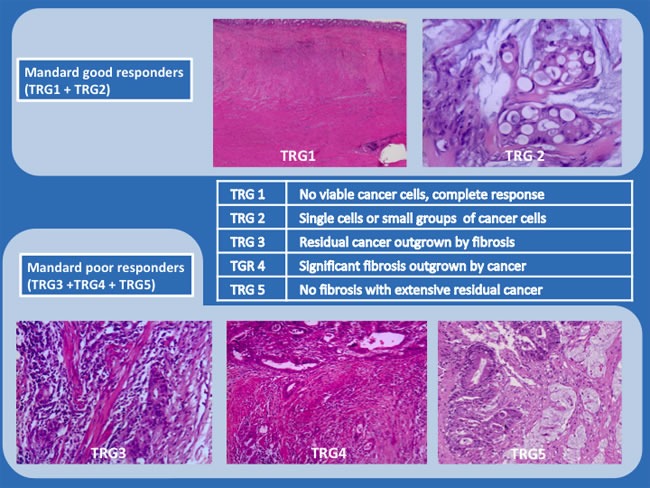
Mandard Tumor Regression Grade System (representative examples; hematoxylin and eosin (H&E) staining; x200 and x100)

Patients were then divided in two groups according to the Mandard TRG system, good responders were defined as Mandard TRG1 orTRG2; poor responders were defined as Mandard TRG3, TRG4 or TRG5 (Figure 3). Both groups (good *versus* poor responders) were used to evaluate outcomes.

### Survival and disease recurrence definitions

Disease recurrence was evaluated according to location: locoregional (LR), systemic (DR), or mixed. All surviving patients were followed-up and their current status was confirmed. None of the patients were lost from follow-up. Disease-free survival (DFS) was calculated from the first date of neoadjuvant treatment to the date of progression (local or distant), and overall survival (OS) was calculated from the first date of treatment to the date of death or last follow-up.

### Statistical analysis

The survival function was estimated using the Kaplan-Meier method. The difference in survival rates between groups was tested for significance using the log-rank test. The significance of differences in proportions was calculated with Chi-square test and the differences in means with Student's t test. A logistic regression analysis was used to assess the independent significance of factors predictive of response, defined as “good” or “poor” responders (TRG1- 2/TRG3-5): age; gender; clinical stage; anal-tumor distance; pre-treatment blood samples of CEA and NLR assay; samples biopsies pre-treatment immunohistochemical expression of cyclin D1, EGFR, VEGF and p21; and 61 tagSNPs were studied. The statistical analysis was made with SPSS statistical software (version 21.0 for Windows; SPSS Inc., Chicago, IL). All statistical tests were conducted at a two-sided level of significance of 0.05.

### Supporting Information

Supplementary Table S1b Description, frequencies and predictive response value of selected SNPs in the 167 patients.

### Availability of data and materials

This is an observational cohort research study with a prospective registration. The data and materials are available in Surgery Department of Hospitalar Center of Porto, Portugal.

## SUPPLEMENTARY MATERIALS TABLE





## Abbreviations

AJCC: American Joint Committee on Cancer
APR: abdominoperineal resection
CEA: carcinoembryonicantigen
Bcl-2: B-cell lymphoma 2
CRM: Circumferential resection margin
COX-2: ciclo-oxigenase 2
CCND1: cyclin D1
EDTA: ethilenediaminetetraacetic
DR: distant recurrence
EGFR: epidermal growth factor receptor
ERUS: endorectal ultrasound
FU: fluorouracil
IHC: immunohistochemistry
LARC: locally advanced rectal cancer
LR: locoregional
NLR: neutrophil lymphocyte ratio
nRCT: neoadjuvant radiochemotherapy
FFPE: formalin fixed paraffin embedded blocks
HE: hematoxylin and eosin
HPGD: Hydroxyprostaglandin dehydrogenase 15-(NAD)
tagSNPs: genomic DNA was extracted from peripheral blood leukocytes or tumor tissue
IGF1: Insulin-like growth factor 1
IL10: interleukin-10
TMA: tissue microarray
TME: total mesorectal excision
TRG: tumor regression grade
VEGF: vascular endothelial growth factor
MRI: magnetic resonance imaging
NAT2: N-acetyltransferase 2
OS: overall survival
PET: positron emission tomography
PGE2: Prostaglandin E2
ROC analysis: receiver operating characteristic analysis
RT: radiotherapy
SNPs: single nucleotide polymorphism
R0: negative margin in resected specimen
R1: cancerous cells on the margin of resected specimen
R2: tumor on the margin of resected specimen even gross examination by the naked eyes
SLCO2A1: solute carrier organic anion transporter family member 2A1
SSRE: sphincter saving rectal resection
TNFα: tumor necrosis factor alpha
ypCR: complete pathological response
