# Supplementary material for: Predictive clinical model of tumor response after chemoradiation in rectal cancer
Source: Oncotarget. 2017 Jul 28;8(35):58133–51. doi: 10.18632/oncotarget.19651 (PMC5601639; doi:10.18632/oncotarget.19651)
Supplement: Supplementary file 1 [file oncotarget-08-58133-s001.pdf]

Predictive clinical model of tumor response after chemoradiation in rectal cancer

SUPPLEMENTARY MATERIALS

For Supplementary Tables see in Supplementary Files
