# Supplementary material for: Predictive clinical model of tumor response after chemoradiation in rectal cancer
Source: Oncotarget. 2017 Jul 28;8(35):58133–51. doi: 10.18632/oncotarget.19651 (PMC5601639; doi:10.18632/oncotarget.19651)
Supplement: Supplementary file 2 [file oncotarget-08-58133-s002.docx]

| **Supplementary Table S1b: Description, frequencies and predictive response value of selected SNPs in the 167 patients.** | | | |
| --- | --- | --- | --- |
| Polymorfism | n | Poor response % | p |
| rs3742106  CC  CA  AA | 29  75  58 | 44.8  45.3  51.7 | 0.72 |
| rs3782958  GG  GC  CC | 122  37  3 | 56.5  59.4  66.6 | 0.18 |
| rs4148421  AA  AG  GG | 41  83  38 | 48.7  46.9  47.3 | 0.98 |
| rs4148422  TT  TC  CC | 109  46  6 | 50.4  41.3  50 | 0.57 |
| rs4148437  TT  TC  CC | 63  83  16 | 48.4  46.9  50 | 0.96 |
| rs4148476  TT  TG  GG | 116  40  6 | 46.5  52.5  33.3 | 0.63 |
| rs4612933  CC  CT  TT | 98  50  13 | 44.8  52  46.1 | 0.71 |
| rs4771912  AA  AG  GG | 120  37  5 | 48.3  51.3  20 | 0.41 |
| rs6492763  TT  TC  CC | 67  73  21 | 47.7  45.2  52.3 | 0.83 |
| rs7993878  GG  GA  AA | 127  33  3 | 46.4  54.5  33.3 | 0.62 |
| rs8002180  TT  TC  CC | 75  67  20 | 44  55.2  35 | 0.19 |
| rs869951  GG  GC  CC | 48  78  33 | 47.9  50  65 | 0.58 |
| rs9524821  GG  GA  AA | 59  86  17 | 42.3  50  52.9 | 0.59 |
| rs9590220  CC  CT  TT | 106  51  6 | 48.1  49  33.3 | 0.76 |

| *ABBC4* gene | | | |
| --- | --- | --- | --- |
| Polymorphysm | n | Poor response % | p |
| rs9590222  GA | 120 | 41.6 | - |
| rs12867485  GG | 163 | 40.4 | - |
| rs1611822  CC  CT  TT | 54  80  29 | 44.4  48.7  51.7 | 0.79 |
| rs1628382  GG  GA  AA | 69  45  5 | 44.9  40  20 | 0.51 |
| rs1678354  TT  TC  CC | 56  83  23 | 48.2  45.7  52.1 | 0.85 |
| rs1678374  TT  TC  CC | 58  86  18 | 48.2  47.6  44.4 | 0.96 |
| rs1678386  AA  AC  CC | 80  63  19 | 51.2  44.4  42.1 | 0.63 |
| rs1678396  TT  TC  CC | 56  83  23 | 48.2  45.7  52.1 | 0.85 |
| rs1678405  TT  TC  CC | 66  88  7 | 43.9  50  42,8 | 0.73 |
| rs1751027  AA  AG  GG | 138  20  4 | 47.1  55  25 | 0.52 |
| rs1751031  AA  AG  GG | 107  49  5 | 45.7  46.9  80 | 0.32 |
| rs1751051  TT  TA  AA | 79  73  11 | 54.4  39.7  54.5 | 0.17 |
| rs2127195  GG  GA  AA | 37  76  35 | 45.9  46  57.1 | 0.51 |
| rs2892713  CC  CT  TT | 102  51  9 | 47  49  44.4 | 0.95 |

| *ABBC4* gene | | | |
| --- | --- | --- | --- |
| Polymorphysm | n | Poor response % | p |
| rs17268122  GG  GT  TT | 93  50  11 | 41.9  54  45.4 | 0.38 |
| rs2274403  AA  AG  GG | 47  84  30 | 51  45.2  50 | 0.78 |
| rs2892715  GG  GA  AA | 57  88  17 | 50.8  44.3  52.9 | 0.66 |

| *SLCO2A1* gene | | | |
| --- | --- | --- | --- |
| Polymorphysm | n | Poor response % | p |
| rs11915399  CC  CT  TT | 115  37  10 | 49.5  45.9  30 | 0.48 |
| rs10935090  CC  CT | 34  9 | 67.6  55,5 | 0.49 |
| rs1131598  AA  AG  GG | 85  67  9 | 48.2  43.2  66.6 | 0.40 |
| rs4241362  TT  TC  CC | 116  41  5 | 50  39  60 | 0.41 |
| rs4241365  TT  TC  CC | 112  38  13 | 49  44,7  46,1 | 0.89 |
| rs4331673  CC  CA  AA | 116  43  3 | 49.1  44.1  33.3 | 0.75 |
| rs4854784  GG  GA  AA | 67  69  24 | 44.7  44.9  62.5 | 0.28 |
| rs6439448  CC  CA  AA | 120  36  6 | 47.5  52.7  33.3 | 0.65 |
| rs7340717  GG  GT  TT | 74  75  11 | 51.3  44  36,3 | 0.51 |
| rs7616492  GG  GA  AA | 64  79  19 | 43.7  46.8  63.1 | 0.32 |

| *SLCO2A1* gene | | | |
| --- | --- | --- | --- |
| Polymorphysm | n | Poor response % | p |
| rs7625035  AA  AG  GG | 98  52  12 | 46.9  42.3  75 | 0.12 |
| rs7646392  CC  CT  TT | 43  87  29 | 37.2  49,4  55,1 | 0.26 |
| rs9820625  AA  AC  CC | 41  80  41 | 46.3  45  56 | 0.49 |
| rs9821091  GG  GA  AA | 72  67  23 | 48.6  43.2  56.5 | 0.53 |
| rs9834412  CC  CA  AA | 96  59  7 | 42.7  50,8  85,7 | 0.073 |

| *HPGD* gene | | | |
| --- | --- | --- | --- |
| Polymorphysm | n | Poor response % | p |
| rs12500316  CC  CT  TT | 98  53  10 | 52  43.3  20 | 0.12 |
| rs1346271  GG  GC  CC | 64  72  26 | 50  50  34 | 0.35 |
| rs1426945  GG  GA  AA | 51  74  36 | 45  55,4  36,1 | 0.14 |
| rs1863642  GG  GT  TT | 86  58  15 | 51.1  44.8  40 | 0.61 |
| rs2303520  GG  GA  AA | 106  50  7 | 44.3  52  71.4 | 0.29 |
| rs2555632  TT  TC  CC | 87  66  9 | 51.7  40.9  55.5 | 0.36 |
| rs8752  TT  TC  CC | 53  81  28 | 50.9  45.6  46.4 | 0.83 |

| *COX2* gene | | | |
| --- | --- | --- | --- |
| Polymorphysm | n | Poor response % | p |
| rs20417  GG  GC  CC | 135  9  11 | 45.9  55.5  45.4 | 0.85 |

| *CCND1* gene | | | |
| --- | --- | --- | --- |
| Polymorphysm | n | Poor response | p |
| rs9344  AA  AG  GG | 53  73  35 | 50  45.2  48.5 | 0.81 |

| *EGFR* gene | | | |
| --- | --- | --- | --- |
| Polymorphysm | n | Poor response | p |
| rs2227983  AA  AG  GG | 8  93  57 | 50  43  52 | 0.30 |

| *IGF1* gene | | | |
| --- | --- | --- | --- |
| Polymorphysm | n | Poor response % | p |
| rs2946834  GG  GA  AA | 75  66  21 | 44  51.5  47.6 | 0.67 |

| *IL10* gene | | | |
| --- | --- | --- | --- |
| Polymorphysm | n | Poor response % | P |
| Rs1800871  GG  GA  AA | 80  67  15 | 38.8  55.2  66.7 | 0.048 |

| *TNFα* gene | | | |
| --- | --- | --- | --- |
| Polymorphysm | n | Poor response % | P |
| rs1800629  GG  GA  AA | 111  46  5 | 51.3  63  60 | 0.22 |

| *Nat2* gene | | | |
| --- | --- | --- | --- |
| Polymorphysm | n | Poor response % | p |
| rs1801280  TT  TC  CC | 95  22  2 | 42.1  40.9  0 | 0.48 |
